# From *A. rhizogenes* RolD to Plant P5CS: Exploiting Proline to Control Plant Development

**DOI:** 10.3390/plants7040108

**Published:** 2018-12-06

**Authors:** Maurizio Trovato, Roberto Mattioli, Paolo Costantino

**Affiliations:** Department of Biology and Biotechnology, Sapienza University of Rome, 00185 Rome, Italy; roberto.mattioli@uniroma1.it (R.M.); paolo.costantino@uniroma1.it (P.C.)

**Keywords:** plant development and organogenesis, proline biosynthesis, RolD, *rol* genes

## Abstract

The capability of the soil bacterium *Agrobacterium rhizogenes* to reprogram plant development and induce adventitious hairy roots relies on the expression of a few root-inducing genes (*rol A, B, C* and *D*), which can be transferred from large virulence plasmids into the genome of susceptible plant cells. Contrary to *rolA*, *B and C*, which are present in all the virulent strains of *A. rhizogenes* and control hairy root formation by affecting auxin and cytokinin signalling, *rolD* appeared non-essential and not associated with plant hormones. Its role remained elusive until it was discovered that it codes for a proline synthesis enzyme. The finding that, in addition to its role in protein synthesis and stress adaptation, proline is also involved in hairy roots induction, disclosed a novel role for this amino acid in plant development. Indeed, from this initial finding, proline was shown to be critically involved in a number of developmental processes, such as floral transition, embryo development, pollen fertility and root elongation. In this review, we present a historical survey on the rol genes focusing on the role of *rolD* and proline in plant development.

## 1. Hairy Roots and *rol* Genes

*Rhizobium rhizogenes*, formerly known as *Agrobacterium rhizogenes* [1,2,3,4,5] is the etiological agent of the hairy root disease, consisting of abundant root proliferation at the site of bacterial infection. The capability of *Rhizobium rhizogenes* to induce hairy roots on susceptible dicotyledonous plants relies on its extraordinary ability to transfer a DNA fragment, called T-DNA, from a large Ri (root-inducing) plasmid to the genome of a plant cell [6,7,8]. The mechanism of T-DNA transfer [9] represents a natural form of genetic engineering, whose comprehension and exploitation has paved the way to the development of plant genetic transformation [10,11,12,13]. 

**Figure 1:**
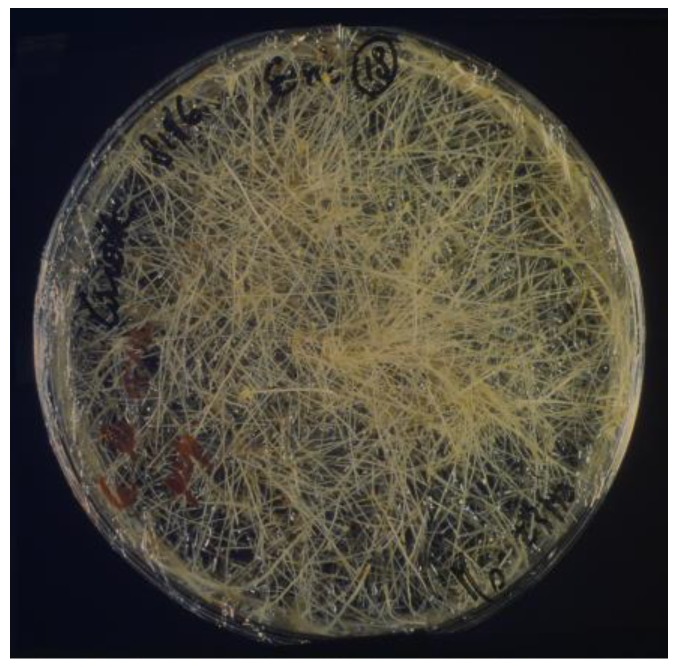
In vitro culture of roots induced on carrot discs by co-inoculation with an *Agrobacterium* strain containing a mannopine-type pRi8196. Once a hairy root culture is established, it can be maintained in vitro without the need of plant hormone supplementation. Fully fertile transgenic plants can be regenerated by these hairy roots.

**Figure 2:**
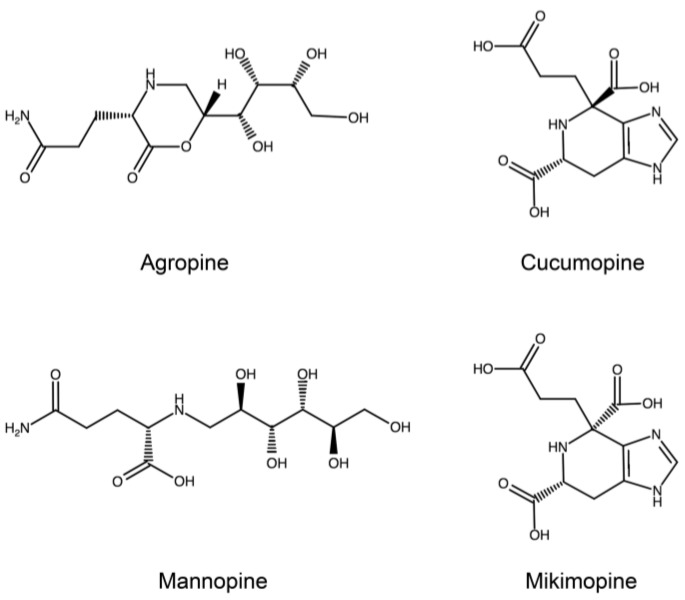
Chemical structure of agropine, cucumopine, mannopine and mikimopine, the four opines found in *A. rhizogenes* strains. The genes responsible for the synthesis of these unusual amino acid-sugar conjugates are borne on the T-DNA, while the genes coding for the catabolic enzymes are found on the non-transferred plasmid portion.

Hairy roots can be easily cultivated in vitro on hormone free medium [14] (Figure 1) and, in most plant species, can also be regenerated into whole fertile plants [15]. In addition, hairy roots produce unusual amino acid-sugar conjugates, called opines (Figure 2) which are not present in normal plant tissues. Depending on the specific Ri plasmid the transforming T-DNA comes from, one of four possible opines, that is agropine, cucumopine, mannopine and mikimopine, is synthesized by enzymes encoded by genes borne on the T-DNA and catabolized by enzymes encoded by genes located on the non-transferred plasmid portion. Because of the tight correlation between the synthesis of a given opine in hairy roots and the utilization of the same opine by the bacterium [16], a further opine-based classification of *Agrobacterium* strains has been proposed and will be adopted in this review. The T-DNA of all the Ri-plasmids have been characterized and sequenced [17,18,19,20]. The T-DNA of cucumopine-, mannopine- and mikimopine-type Ri plasmids turned out to consist in a continuous stretch of DNA, while the T-DNA of the agropine-type Ri plasmid is split in two T-DNA, called TR- and TL-DNA, which are independently transferred and integrated into the plant cell. Subsequent genetic work has clearly shown that the TL-DNA is uniquely responsible for hairy root induction, while the TR-DNA plays an accessory role to facilitate hairy root induction in some recalcitrant plant species. In a seminal work by White et al. [21] an extensive mutagenesis analysis was carried out, by transposon tagging, on the agropine-type pA4 plasmid. The genetic analysis led to the identification of four classes of mutations capable to affect the rooting phenotype and denominated, accordingly, *rol* (*ro*ot *l*oci) *A*, *B*, *C* and *D*. To further identify their functions, different *rol* combinations were cloned into binary vectors and transferred to *Agrobacterium* [22] to be used either for infection experiments on different plant hosts or for generating transgenic plants. The first analyses confirmed that the rol genes were the only Ri T-DNA segments responsible for hairy root induction and showed that a DNA fragment encompassing *rolA*, *B* and *C* was almost as effective in inducing hairy roots as the whole Ri T-DNA [23]. Accordingly, because of the functional importance of *rolA*, *B* and *C* and because these genes are present in all virulent strains of *Agrobacterium rhizogenes*, most of the studies initially focused on these oncogenes, particularly on *rolB*, while little attention was paid to *rolD*. Most of the aspects related to *Agrobacterium*, hairy roots and *rolA*, *B* and *C*, have been covered by excellent reviews [24,25,26] and will not be further expanded.

## 2. RolD

As already noted, *rolD* is not present in all virulent *A. rhizogenes* strains and therefore plays a marginal role in hairy root induction. However, transformation experiments [27,28,29] showed that expression of *rolD* is developmentally regulated in and can deeply affect the development of plant cells. Tobacco plants expressing *rolD* driven by its own promoter have been reported to reach anthesis in average 60 days (in some cases as many as 75) before untransformed plants [28]. The inflorescence was richer and long-lasting, compared to controls plants and the overall morphology of the plants was deeply altered, with a strong reduction in height and with tiny and bract-like leaves. Furthermore, organogenesis experiments on thin cell layers (TCL) from *rolD* and control plants cultured on different synthetic media confirmed and extended in vitro the notion that *rolD* has the potentiality to enhance and anticipate flower formation [28]. Similar results were obtained in tomato [30] and *Arabidopsis* [31]. The small size typical of all *rolD*-expressing transgenic plants, may be accounted for by the early and abundant proliferation of axillary buds, leading to highly branched shoots. Down-regulation of *CYP79F1*/*SUPERSHOOT*/*BUSHY* (*SPS*), a gene involved in glucosinolate biosynthesis [32], was reported in *Arabidopsis* transformed with *rolD* [31]. Since *SPS* normally inhibits the formation of lateral shoots by altering cytokinin balance, the proliferation of axillary branches of Arabidopis transgenic for *rolD* may be accounted for by a (secondary) effect of *rolD* on *SPS* expression. 

It is not clear how *SPS* downregulation can affect the cytokinin/auxin ratio, since the synthesis of indole glucosinolates in *Arabidopsis* proceeds from the transformation of tryptophane to indole-3-acetaldoxime catalysed by CYP79B2 and CYP79B3 [33], while CYP79F1 is involved in the biosynthesis of aliphatic glucosinolates [32,34]. However, CYP79F1/SPS has the potentiality to affect cytokinin/ auxin balance through the synthesis of a common aldoxime precursor. Consistently, a null *CYP79F1* mutant (*bus1-1f*), totally devoid of short-chain methionine-derived glucosinolates, was also found enriched in indole-3-methyl-glucosinolate, indole-3-acetic acid and indole-3-acetonitrile [34].

Histochemical analysis of tobacco plants expressing the GUS reporter gene driven by the *rolD* promoter, revealed that this gene has a complex pattern of expression under strict developmental control [29]. Unlike other *rol* genes, which are always expressed in meristematic tissues, the promoter of *rolD* is not active in plant meristems but rather works in all growing and differentiating tissues throughout development, from the embryo to the adult plant. In particular, the expression of *rolD* characterizes the region of elongation and expansion of every tissue and organ. Intriguingly, as already mentioned, mutations in *rolD* prevent the T-DNA-induced hairy roots from elongating after initiation [21]. This suggests the possibility that *rolD* may be functionally involved in the process of elongation and/or maturation of roots and, possibly, of other organs. 

**Figure 3:**
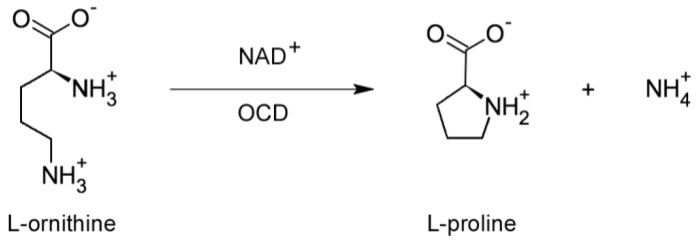
Proline synthesis from ornithine. The enzyme ornithine cyclo deaminase (OCD), an enzyme frequently found in bacteria but uncommon in plants, catalyses the NAD^+^-dependent conversion of ornithine into proline and NH_4_^+^.

A similarity search, based on a combination of iterative and noniterative methods, detected a highly significant sequence similarity between *rolD* and the gene coding for ornithine cyclodeaminase (OCD), an unusual enzyme of bacterial origin that catalyses the direct conversion of ornithine and NAD+ into proline and NH_4_^+^ [35] (Figure 3). This bioinformatic prediction was experimentally confirmed by enzymatic assays on RolD expressed and purified in *E. coli* and on soluble extracts from plants overexpressing the oncogene under the control of a CaMV35S promoter. The enzymatic assays revealed a specific ornithine-dependent proline production, associated to NAD+ reduction, that could only be accounted for by OCD activity. No functional OCDs have been detected so far in plants [36], where ornithine is converted to proline only via pyridoxal phosphate-dependent reactions. OCD seems to be a specialized enzyme that has been found only in a limited number of prokaryotic species, such as *Agrobacterium*, *Sinorhizobium*, *Rhodobacterium* and *Brucella* as well as in some extremophile archaea, such as *Archeoglobus* and *Methanobacterium*, where it is involved in the catabolism of unusual carbon and nitrogen sources like opines or methane. Interestingly, in *A. tumefaciens* OCD is encoded by genes localized in the non-transferred part of the Ti plasmid [7,37] to be used for opine catabolism, while in *A. rhizogenes*, OCD has become part of the T-DNA and it is expressed only in the plant cells. Intriguingly, in animals the mu-crystallins family of proteins shares significant similarities with OCD. This is not surprising, because in the mammalian eye often lens proteins derive from metabolic enzymes or stress proteins, which acquire reflective properties while, in some cases, maintaining their original metabolic activity [38]. 

Since *rolD* is only present in the TL-DNA of the agropine-type Ri plasmids, its expression seems not strictly required for hairy root elongation. Although not experimentally demonstrated, it is tempting to speculate that *rolD*, similarly to the ancillary role played by the T_R_-DNA-borne *iaaH* and *iaaM* genes in the process of hairy root induction, might play an auxiliary role in hairy root elongation by providing more proline in hosts with low levels of endogenous proline or during environmental stresses requiring higher proline demand. In support of this hypothesis, proline has been shown to accumulate during the elongation of the maize primary roots at low water potential [39].

As alternative explanation, other genes, either belonging to the Ri T-DNA or to the plant genome itself, could functionally substitute for *rolD* expression. This hypothesis is based on the work of Levesque et al (1988) [40] who observed that the Ri TL-DNA genes are functionally redundant and may derive from a common ancestral T-DNA. Redundancy, according to authors, would serve as an adaptive strategy to ensure function in a variety of host species and environmental conditions [40]. In the case of *rolD*, a recent duplication has apparently occurred between ORF 15 (*rolD*) and the ORFs 18 and 17, which, assembled together, restore a direct repetition of *rolD* [40]. Furthermore, portions of the Ri TL-DNA plasmid, including *rolD*, have been detected in the genome of some plant species (ct-TDNA), probably as a result of ancient *Agrobacterium* transformations [41,42]. It must be noted that, since OCD activity, in addition to proline, also produces NH4, a major nitrogen source which behaves as a signalling molecule capable of triggering multiple physiological and morphological responses in plants [43], we cannot rule out the possibility that some of the developmental alterations attributed to OCD may be accounted for, or contributed to, a perturbed ammonium homeostasis. 

## 3. The Role of Proline in Plant Development 

The discovery that RolD is a proline-synthesizing enzyme involved in root elongation [21,29] but also in flowering time [21,28,30], implied the possibility that this cyclic amino acid may have a role in plant development. It was already well-established that proline, in addition to its role in protein synthesis, is involved in the plant cell response to many types of stresses, essentially because a strong proline accumulation is observed soon after stress occurrence in many plant species [44].

However, proline accumulation was also described, in non-stressed conditions, in the tissues and organs of different plant species, particularly during the reproductive phase [45,46,47,48,49,50], supporting the idea that proline may play a role in plant reproductive development in normo-osmotic conditions. In the total amino acid pool of *Arabidopsis*, the percentage of proline raises from 1–3% in vegetative tissues before floral transition, to 26% in reproductive tissues after floral transition [49]. Similarly, Schwacke et al. (1999) [50] observed that the content of free proline in tomato flowers was 60-fold higher than in any other organ analysed. Although proline is a relatively common amino acid in plants, because of the frequent occurrence of long stretches of proline or hydroxyproline residues in cell wall proteins, particularly extensins [51], it is unlikely that, in non-stressed conditions, such large amount of proline can be accumulated for the needs of protein synthesis. 

Differently from OCD, which catalyses the direct conversion of ornithine to proline, in higher plants proline is mainly synthesized in the cytosol from glutamate in a two-step reaction involving the enzyme δ-pyrroline-5-carboxylate synthetase (P5CS) and δ-pyrroline-5-carboxylate reductase (P5CR). Subsequently, proline is exported to the mitochondrion where it is catabolized back to glutamate by the enzymes proline dehydrogenase (ProDH) and δ-pyrroline-5-carboxylate synthetase P5CDH [44]. An alternative route starting from ornithine and mediated by ornithine δ-aminotransferase (δOAT) has also been reported [52], at least in some physiological conditions but its functional significance in maintaining proline homeostasis is strongly controversial [53,54]. 

The genes coding for the anabolic and catabolic enzymes of proline synthesis are highly conserved among plant species, although *P5CS* and *ProDH*, the genes coding for the rate-limiting steps of the anabolic and, respectively, catabolic pathways, may be present in multiple variants [55]. In *Arabidopsis*, P5CS is encoded by two paralog genes *P5CS1* and *P5CS2* [56], whose deduced amino acid sequences share 98% amino acid identity. In spite of the high similarity of these isoforms, *P5CS1* and *P5CS2* have a different tissue specificity and play non-redundant but partially overlapping functions, as inferred by the analysis of transgenic *Arabidopsis* carrying mutations in either *P5CS1* or *P5CS2* [57,58]. *P5CS1* is responsive to stress induction, while *P5CS2* is constitutively expressed at low levels in all tissues and organs and at high level in meristematic tissues, floral organs and in embryos [57,58].

### 3.1. Floral Transition

**Figure 4:**
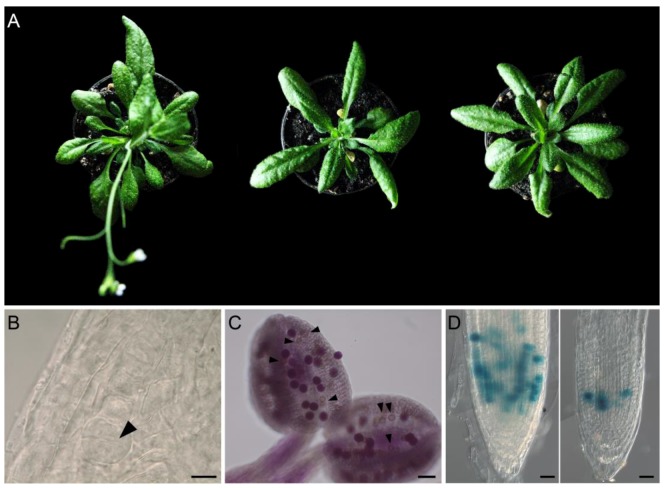
Effects of proline on plant development. (**A**) *Arabidopsis p5cs1* mutants (middle) and to a greater extent, *p5cs1 p5cs2/P5CS2* sesquimutants (right), defective in proline synthesis, are late flowering, compared to a wildtype (left) [68]. (**B**) Aberrant orientations of cellular division planes observed in an octant embryo from a segregating population of heterozygous *p5cs2*/+ [58]. (**C**) *Arabidopsis* anthers from *p5cs1 p5cs2/P5CS2* sesquimutants stained with the vital Alexander’s staining show a population of stained and viable pollen grains mixed with a population of unstained and unviable aberrant pollen grains (indicated by arrows) [69,79]. (**D**) GUS staining of *CYCB:GUS* roots in a wildtype (leftmost side) and a *p5cs1 p5cs2/P5CS2* (rightmost side) background reveals the effects of proline on cell division [80]. Arrows show the aberrant division planes in an octant embryo in (B) and the unstained, unviable pollen grains in a *p5cs1 p5cs2/P5cs2* anther in C. Bars = 10 µm (B), 50 µm (C) and 20 µm (D).

Consistent with the strong anticipation and stimulation of flowering induced by the ectopic expression of *rolD* [28,30,31], a number of authors reported, in absence of stressing stimuli, upregulation of both proline biosynthesis (*P5CS, P5CR*) and transport genes (*ProT*) in reproductive tissues [50,59,60], such as flowers, inflorescences and anthers, suggesting a possible role of proline in flowering. Intriguingly, the expression of the proline catabolic genes (*ProDH, P5CDH*) was also reported to increase in reproductive tissues under normo-osmotic conditions [61,62,63], in striking contrast with the strong downregulation of these genes observed under stressed conditions [64,65]. In agreement with these data, Kavi Kishor et al. (1995) [66] reported that constitutive overexpression of *P5CS1* in tobacco plants enhances flower development under drought conditions, while Nanjo et al. (1999) [67] reported that antisense expression of *P5CS1* inhibits bolting in *Arabidopsis*. *Arabidopsis p5cs1* mutants and to a greater extent, *p5cs1 p5cs2/P5CS2* sesquimutants, exhibited a strong delay in floral transition [58,68,69] (Figure 4A), while transgenic *Arabidopsis* overexpressing *P5CS1* under the control of the strong and constitutive CaMV35S promoter, showed a striking anticipation of flowering time and a proliferation of coflorescences, particularly in short day conditions [68]. In transgenic *35S::P5CS1* plants, the expression of the recombinant *P5CS1* was downregulated after flower transition, along with the endogenous allele of *P5CS1* and *P5CS2*, and, accordingly, *P5CS1* was overexpressed only for a short time, up to floral transition [68]. Altogether, these data suggest that proline plays a key role in flower transition, bolting and coflorescence architecture. 

Presently, the molecular mechanism through which proline affects flowering time is not clear but it seems quite different from the mechanism through which proline protects plant cells from stress injuries [70]. One major difference between these mechanisms is the concentration of proline involved: during floral transition proline reaches only a localized and transient increase in the shoot apical meristem (SAM), while under stress conditions, it accumulates at high levels in all the tissues of the plant [68]. The accumulation of proline measured in *35S-P5CS1* plants (up to 3-fold the level of the wildtype), seems modest compared to that achieved under stress, where proline levels are 10 to 20-fold higher than in unstressed plants [68], suggesting that this amino acid may behave as a floral signal able to interact with flower regulators. It is well known that floral transition, i.e., the transition from a vegetative shoot apical meristem (SAM) to a floral SAM, involves a profound change in the identity of the apical meristem that starts producing flowers rather than leaves [71]. This switch to vegetative to reproductive development is regulated by a number of environmental and endogenous inputs, which converge to regulate master flowering regulators, which, in turn, activate floral identity genes. By genetic analysis four major pathways have been identified, photoperiodic, autonomous, vernalisation and gibberellin pathway [72,73,74], which are controlled by the master regulators *CONSTANS* (*CO*) and *FLOWERING LOCUS C* (*FLC*), which, in turn, control the floral integrators *LEAFY* (*LFY*), *SUPPRESSOR OF CONSTANS 1* (*SOC1*) and *FLOWERING TIME* (*FT*) to eventually activate the floral identity genes *APETALA 1* (*AP1*), *APETALA 2* (*AP2*), *FRUITFULL* (*FUL*) and *CAULIFLOWER* (*CAL*). In agreement with the hypothesis that proline may behave as a floral signalling molecule, *P5CS2* has been identified as an early regulatory target of *CONSTANS* (*CO*), a master transcriptional regulator of the photoperiodic pathway [75]. However, because of the importance of proline as redox buffer [76] and ROS scavenger [77], we cannot rule out the possibility that proline may act as an active metabolite involved in metabolic signalling [78]. Overall, the body of accumulated evidence points to proline as a modulator of floral transition although a full comprehension of its mechanism of action and of the floral pathway it interacts with is still to be gained.

### 3.2. Embryo Development

Proline seems to play an important role also in plant embryogenesis. Quite surprisingly, despite the high sequence similarity shared by *P5CS1* and *P5CS2* and although both genes share the same pattern of expression in shoot apical meristems and embryos [57,58], *p5cs2* but not *p5cs1* mutants, are embryo lethal suggesting a specific role of *P5CS2* in embryogenesis. The embryo defects (Figure 4B) can be partially [57,58] or totally [69] complemented by treatment with L-proline suggesting that mutations in *P5CS2* specifically affect proline accumulation in developing seeds. The reason for such striking differences between P5CS1 and P5CS2 are not fully understood but it may be related to different subcellular localization of these two proteins in the embryo, as proposed by Szekely et al (2008) [57] who detected a P5CS1:GFP fusion protein outside the cytoplasm within subcellular bodies, while P5CS2:GFP had a cytoplasmic localization. A microscopic analysis of the mutant embryos revealed a number of aberrations typically associated with defects in cell cycle progression, such as anomalous orientations of the cellular division planes (Figure 4B), multi-nucleate suspensor cells and adventitious embryos [58], suggesting a possible relation between proline and cell cycle. 

### 3.3. Pollen Fertility

One the best-known and less-explained fact on pollen composition is the exceedingly large amount of proline found in different plant species [49,50,81,82,83] suggesting a special role for proline in pollen development and function. At present, it is not known how proline can accumulate in pollen in such large quantities. In principle, proline could be synthesized inside gametophytic pollen grains, in surrounding sporophytic tissues, such as the tapetum or the intermediate layer, or be transported through phloem or xylem vessels from far away tissues. Because single, double and triple knockout mutants for all the genes belonging to the AtProT family—the best known group of proline transporter in plants—showed no differences, compared to wildtype, [84] and because microarray data detect strong expression of proline biosynthesis genes in pollen and anthers [85], proline accumulation in pollen grains likely derive from endogenous synthesis either in sporophytic or in gametophytic tissues of the anther. 

Since pollen grains are subjected to a process of natural dehydration during their maturation, a role for proline as a compatible osmolyte has been proposed by some authors [49], while others [86] postulated that this amino acid may act as source of energy or metabolic precursor to fuel the rapid elongation of the pollen tube. A sound scientific base to settle this contrasting views was independently given by two research groups [69,79] who demonstrated, by a combination of genetic and physiological experiments, that a *p5cs1 p5cs2/P5CS2* sesquimutant, homozygous for *p5cs1* and heterozygous for *p5cs2*, was strongly impaired in pollen fertility [87]. 

The fertility defects of the sesquimutants were accounted for by defects in pollen grains, a number of which-presumably those with a *p5cs1*, *p5cs2* haploid genotype-were degenerated and unviable (Figure 4C). The proline content of the sesquimutant pollen population was measured and found to be less than a third compared to wildtype pollen. Moreover, exogenous proline supplied from the beginning of anther development was shown to partially complement both morphological and functional defects of the aberrant pollen grains. All in all, these data indicate that proline is required for pollen development and fertility and further corroborate the notion of the crucial importance of proline in reproductive development.

### 3.4. Root Elongation

In addition to its role in plant reproductive development, a novel role as modulator of root growth has been recently ascribed to proline [80]. In plants, postembryonic root growth is driven by the activity of the root meristem, which continuously regenerates itself in the staminal niche, while generating transit-amplifying cells, which undergo additional division in the proximal meristem and eventually, differentiate in the meristem transition zone. The balance between cell proliferation and cell differentiation determines root meristem size and, in turn, root growth and is largely controlled by plant hormones, particularly cytokinin and auxin [88].

The size of the root meristem, expressed as the number of cortex cells spanning from the quiescent centre (QC) to the first elongated cell in the transition zone (TZ) [89], was analysed in *p5cs1 p5cs2/P5CS2* sesquimutants relative to wildtype. Proline-deficient mutants were found to have root meristems remarkably smaller than the wildtype and the addition of micromolar concentrations of exogenous proline fully rescued the sesquimutant root meristem to wildtype size. Importantly, the effect of exogenous proline was also tested on wildtype roots and shown to have a specific and dose-dependent stimulatory effect at low concentrations and an inhibitory effect at high concentrations [80].

Considering the role played by *rolD* in the hairy roots syndrome, it not surprising that proline can modulate root elongation. Indeed, in the genesis of hairy roots RolD/OCD is involved in the elongation of roots generated by the combined action of RolA, B and C [21]. In addition, exogenous proline at micromolar concentration was shown to induce elongation of both primary and secondary roots in Arabidopsis [58]. 

The action of proline on root meristem seems independent of the plant hormones auxin, cytokinin and gibberellin as shown by a combination of pharmacological, molecular and genetic experiments [80]. Proline was found to regulate cell division in early stages of root development modulating the expression of *CYCB1;1*, the gene coding for the G2/M-specific CYCLINB1;1 (Figure 4D).

Other hormone-independent mechanisms are known to modulate root growth, such as the superoxide/hydrogen peroxide ratio reported by Tsukagoshi et al. (2010) [90] but the case of proline is quite surprising because the accumulation of this amino acid in the root is under strict abscisic acid (ABA) control under stress conditions [91]. However, proline has also been shown to be regulated by non-ABA-dependent factors [60,92] and it is possible that two parallel signalling pathways can independently control proline-dependent root regulation under stressed and, respectively, non-stressed conditions.

## 4. Conclusions and Perspectives

Much like the hairy root syndrome, which was originally thought as a simpler variant of the crown gall disease but eventually turned out to be a highly sophisticated and, as yet, not fully understood biological mechanism, the role of proline in plant development is unveiling unexpected complexities in plant development. Thanks to the study of *rolD*, we now know that proline can modulate the size of the root meristem independently of plant hormones and finely tune development in reproductive organs, although we are still far from a full comprehension of the underlying mechanisms of action. In a way, much like plant hormones, proline may behave as a second messenger. Because of the remarkable chemical-physical properties of this cyclic amino acid, however, a mechanism mediated by or dependent on metabolic regulations cannot be ruled out. 

The long scientific journey from hairy roots to RolD to plant P5CS has produced more open questions than definitive answers. Our understanding of the genetic and molecular mechanisms through which proline exerts its effects on plant development is still rudimentary. We do not know whether the effects of proline on different developmental processes are mediated by different mechanisms or share a common molecular machinery. Clearly, further work is needed to fully understand the complex molecular mechanism/s by which proline can finely tune developmental processes as diverse as hairy root elongation, floral transition or pollen fertility.
